# Bioactive Materials for Bone Tissue Engineering

**DOI:** 10.1155/2016/3741428

**Published:** 2016-01-28

**Authors:** Julietta V. Rau, Iulian Antoniac, Giuseppe Cama, Vladimir S. Komlev, Antonio Ravaglioli

**Affiliations:** ^1^Istituto di Struttura della Materia-Consiglio Nazionale delle Ricerche (CNR-ISM), Via del Fosso del Cavaliere, 100-00133 Rome, Italy; ^2^Biomaterials Group, Materials Science and Engineering Faculty, University Politehnica of Bucharest, Splaiul Independentei 313, Building J, Room JK109, Sector 6, 060032 Bucharest, Romania; ^3^Polymer Chemistry & Biomaterials Group, Krijgslaan 281 (S4-Bis), 9000 Ghent, Belgium; ^4^A. A. Baikov Institute of Metallurgy and Materials Science, Russian Academy of Sciences, Leninsky Prospect 49, Moscow 119991, Russia

For early biomaterials, it was required to have a combination of physicochemical properties, suitable to replace human body tissues and to be biologically inert. Since then a long way has been brought to the third generation of biomaterials, whose role is to be both resorbable and bioactive, that is, to be able to elicit a controlled action in physiological conditions. Recently, the advances in cellular proteomics and genomics paved the way to the fourth generation of biomaterials, known as biomimetic and smart materials, and to their application in regenerative medicine.

There is a wide range of materials used for orthopaedics and dentistry, from metals, alloys, ceramics, and polymers to bioactive glass systems and hybrid composites. It is very important to know the performance of these materials used in clinical practice or of some newly developed materials, starting from* in vitro* tests to the* in vivo* animal models.

This issue is focused on the new frontiers in research studies dedicated to the use of bioactive materials in different forms, like scaffolds, composites, or coatings, for biomedical applications and related clinical investigations. Based on the requirements of the modern biomedical technology, the novel research strategies in biomaterials field are nowadays directed towards biomaterials endowed with surface properties and characteristics suitable for drug delivery and for the controlled release of active principles, especially against infections.

In this view, several research groups have been invited to contribute to this special issue with their original research and review papers that could stimulate efforts of comprehensive knowledge of bioactive materials with clinical applications in bone tissue engineering. This special issue is divided into three categories based on the following keywords: bioactive materials performance, smart materials and technologies for bone tissue engineering applications, bioactive coatings for osseointegration, and antimicrobial activity.

In the category of bioactive materials performance, calcium phosphate and bioactive glass based materials have attracted a significant attention being widely used in bone tissue engineering. In particular, calcium phosphates are more traditional for bone graft substitution, since their composition is close to the mineral part of the bone tissue. However, their performances could still be improved.

I. Ajaxon et al. evaluated the long-term* in vitro* degradation of high-strength brushite calcium phosphate cements over a time period of 25 weeks in water, phosphate buffered saline, and a serum solution. The compressive strength, chemical composition, and microstructure were also evaluated, and important changes in the properties of the cements were observed after 10 weeks of incubation time.

Y. Ayukawa et al. evaluated the tissue/cellular response toward the low-crystalline carbonate apatite (CO_3_Ap), the form of apatite found in bone, which was fabricated using a dissolution-precipitation reaction with set gypsum as a precursor. Both CO_3_Ap and sintered hydroxyapatite, used as a control, were implanted into surgically created tibial bone defects of rats for histological evaluation. Authors observed that the CO_3_Ap granules mimicked the bone matrix, suggesting that CO_3_Ap may be an appropriate bone substitute.

B. Sarikaya et al. developed porous collagen/*β*-tricalcium phosphate (TCP) based scaffolds with superior mechanical properties. The scaffolds obtained via dehydrothermal processing are able to bend and be rolled, making them suitable for a wide range of handlings. This study suggested that the cross-linked collagen/*β*-TCP scaffolds show great potential for use in bone tissue engineering applications.

W. P. Chan et al. described the technique for synthesizing of biocompatible alginate/poly(*γ*-glutamic acid) base gel with potential application as injectable bone repair material. Evaluation of its mechanical properties, swelling behaviour, and blood compatibility showed the nontoxicity of this material and that it could be used for repairing of bone defects.

Concerning bioactive glasses, the focus point is their ability to continuously exchange ions with physiological liquids, releasing appropriate trace elements in order to stimulate specific cellular response, aimed at activating genes responsible for osteogenesis and bone tissue regeneration.

N. A. P. van Gestel et al. provided an overview on S53P4 bioactive glass current clinical results in such applications as craniofacial reconstructions, grafting of benign bone tumour defects, instrumental spondylodesis, and the treatment of osteomyelitis, whereas D. Bernardeschi et al. evaluated the cutaneous and the inner ear tolerance of S53P4 bioactive glass when used in the mastoid and epitympanic obliteration for chronic otitis surgery. Bioactive glass granules resulted to be well tolerated for primary or revision chronic otitis surgery.

In the category of smart materials and technologies for bone tissue engineering applications, the advances in cellular proteomics and genomics paved the way to the smart materials and to their application in regenerative medicine.

F. Wang et al. investigated the osteogenic capacity of nano-hydroxyapatite/chitosan/poly(lactide-co-glycolide) (nHA/CS/PLGA) scaffolds seeded with human umbilical cord mesenchymal stem cells in the calvarial defects of the nude mice. It was found that nHA and CS enhance the bone regeneration at early stage, opening the way to the scaffold applications in bone tissue engineering.

C. Romagnoli et al. analysed the behaviour of a clonal finite cell line derived from human adipose tissue seeded on poly(*ε*-caprolactone) film, prepared by solvent casting. The adipose-derived mesenchymal stem cells (AMSCs) were able to adhere to the biomaterial and remained viable for the entire experimental period. Moreover, the proliferation process and osteogenic activity of AMSCs were maintained on the biofilm, demonstrating that the selected biomaterial ensures cell colonization and the development of an extracellular mineralized matrix.

N.-H. Kim et al. evaluated the effect of rhBMP-2 (recombinant human bone morphogenetic protein) applied in different concentrations to sandblasted and acid etched titanium implants, in bone defects of beagle dogs using split-mouth design. The authors reported that titanium implants coated with rhBMP-2 were more effective, compared with untreated group, in promoting bone regeneration and osseointegration.

R. H. Pedersen et al. reported the efficacy of inorganic bone mineral coated with P-15 peptide (ABM/P-15) on tibia defect repair longitudinally in both normal and osteoporotic rats* in vivo*. It was shown that the ABM/P-15 accelerated bone regeneration in osteoporotic rats but did not enhance bone regeneration in normal rats.

New advanced technologies could be used to process bioactive materials in order to obtain scaffolds or coatings for biomedical applications.

D. Du et al. considered the problem of shape matching between bone defects and ceramic scaffolds. Computer-assisted microstereolithography (MSTL) can fabricate anatomically shaped complex three-dimensional structures. However, thermal contraction mismatch between the scaffold material and mold results in shape distortion during sintering. The authors developed a novel gelcasting indirect MSTL technology to overcome this problem and successfully fabricated beta-tricalcium phosphate scaffolds for rabbit femur. The reported fabrication method could be useful for constructing bone substitutes specifically designed according to local anatomical defects.

M. B. Bezuidenhout et al. reviewed the studies related to the titanium-based hip stems with drug delivery functionality through additive manufacturing, starting from the idea that the treatment of infected femoral stems is a major problem in clinical practice and remains an area of debate. Through additive manufacturing, antibiotics can be incorporated into cementless femoral stems to produce prosthetic devices with antimicrobial properties. The review highlighted the microorganisms associated with total hip arthroplasty, discussed the advantages and disadvantages of the latest materials used in hip implants, compared different antimicrobial agents that could be incorporated, and addressed novel ideas for future research.

In the category of bioactive coatings for osseointegration and antimicrobial activity, the novel research strategies in biomaterials field are nowadays directed towards biomaterials endowed with surface properties and characteristics suitable for drug delivery and for the controlled release of active principles, especially against infections.

With this regard, C. S. Ciobanu et al. reported the preparation and characterisation of hydroxyapatite doped with silver/polydimethylsiloxane composite layer and its antimicrobial effect against* Candida albicans* biofilm embedded cells.

S. Yeniyol et al. reported the preparation and investigation of anatase/rutile mixed-phase TiO_2_ thin films on commercially pure Ti sheets and their photocatalytic activity for possible antibacterial use around dental implants. The photocatalytic performance against* A. actinomycetemcomitans* colonization was testified.

R. A. Garcia et al. evaluated the polymethyl methacrylate and calcium sulfate as carriers for the local delivery of gallium (III) nitrate (Ga(NO_3_)_3_), which were reported to exert a broad antimicrobial activity against organisms commonly associated with orthopaedic related infections. The* in vitro* findings suggested that local delivery of Ga(NO_3_)_3_ may be an effective strategy for the prevention and/or treatment of orthopaedic related infections. The authors concluded that future studies utilizing animal models are needed to fully characterize the clinical role for this treatment modality.

Apart from microstructure and composition characteristics, a special stress should be given to the host tissue/material interface proven by the* in vivo* performance.

H.-K. Tsou et al. reported highly osteoblast compatible titanium dioxide (TiO_2_) coatings on polyetheretherketone (PEEK). After TiO_2_/PEEK prolonged implantation in rabbits, histological observation showed newly regenerated bone formed more prominently. The shear strength of the bone/implant interface increased with the increase of implantation period. Bone bonding performance of the TiO_2_/PEEK implant was superior to that of the bare PEEK. The rutile-TiO_2_ coatings achieved better osseointegration than the anatase-TiO_2_ coatings.

By collecting these papers, we hope to enrich our readers and researchers in the field of bioactive materials for bone tissue engineering. We believe that novel bioactive materials will be an important part of future bone tissue engineering products.

